# Assessment of the quality of DNA from various formalin-fixed paraffin-embedded (FFPE) tissues and the use of this DNA for next-generation sequencing (NGS) with no artifactual mutation

**DOI:** 10.1371/journal.pone.0176280

**Published:** 2017-05-12

**Authors:** Naoki Einaga, Akio Yoshida, Hiroko Noda, Masaaki Suemitsu, Yuki Nakayama, Akihisa Sakurada, Yoshiko Kawaji, Hiromi Yamaguchi, Yasushi Sasaki, Takashi Tokino, Mariko Esumi

**Affiliations:** 1 Department of Pathology, Nihon University School of Medicine, Itabashi-ku, Tokyo, Japan; 2 Department of Orthopaedic Surgery, Nihon University School of Medicine, Itabashi-ku, Tokyo, Japan; 3 Department of Oral Pathology, Nihon University School of Dentistry at Matsudo, Matsudo, Chiba, Japan; 4 Department of Medical Genome Sciences, Sapporo Medical University, Sapporo, Hokkaido, Japan; Queen Mary Hospital, HONG KONG

## Abstract

Formalin-fixed, paraffin-embedded (FFPE) tissues used for pathological diagnosis are valuable for studying cancer genomics. In particular, laser-capture microdissection of target cells determined by histopathology combined with FFPE tissue section immunohistochemistry (IHC) enables precise analysis by next-generation sequencing (NGS) of the genetic events occurring in cancer. The result is a new strategy for a pathological tool for cancer diagnosis: ‘microgenomics’. To more conveniently and precisely perform microgenomics, we revealed by systematic analysis the following three details regarding FFPE DNA compared with paired frozen tissue DNA. 1) The best quality of FFPE DNA is obtained by tissue fixation with 10% neutral buffered formalin for 1 day and heat treatment of tissue lysates at 95°C for 30 minutes. 2) IHC staining of FFPE tissues decreases the quantity and quality of FFPE DNA to one-fourth, and antigen retrieval (at 120°C for 15 minutes, pH 6.0) is the major reason for this decrease. 3) FFPE DNA prepared as described herein is sufficient for NGS. For non-mutated tissue specimens, no artifactual mutation occurs during FFPE preparation, as shown by precise comparison of NGS of FFPE DNA and paired frozen tissue DNA followed by validation. These results demonstrate that even FFPE tissues used for routine clinical diagnosis can be utilized to obtain reliable NGS data if appropriate conditions of fixation and validation are applied.

## Introduction

Due to the rapid progress in next-generation sequencing (NGS), cancer genomics is revealing the somatic variants and driver mutations of genes in various cancers [1, 2]. In particular, formalin-fixed, paraffin-embedded (FFPE) tissues used for pathological diagnosis are valuable for studying cancer genomics. Compared with fresh-frozen tissue samples, FFPE samples have the following 4 advantages for applications in cancer genomics. 1) FFPE tissue samples allow for a retrospective study, with increases in the number of cancer cases and types. 2) FFPE sections displaying various histological features of cancer, including precancerous lesions, enable assessment of the genetic events related to the observed histological changes. 3) When dissecting cancer evolution, more precise analysis of genetic events can be achieved with laser-capture microdissection (LCM) of target cells from FFPE sections. 4) Immunohistochemistry (IHC) of FFPE sections helps in extracting specific target cells by LCM to elucidate genetic alterations in specific marker-positive cells. Such combination technology, i.e., FFPE/LCM/NGS, termed ‘microgenomics’, is helpful for developing new genetic biomarkers and a new pathological tool for cancer diagnosis.

NGS analysis of FFPE DNA has great potential for expanding microgenomics. However, DNA extracted from FFPE tissues has some limitations for genomic analysis due to the possibility of DNA fragmentation and cross-links by chemical modification [3]. Thus, to precisely clarify the limitations of using FFPE DNA for NGS and to improve DNA quality as much as possible, we investigated the following three issues regarding FFPE DNA. 1) To determine the most appropriate conditions for FFPE DNA, we performed systematic analysis of formalin fixation, such as formalin concentrations and incubation durations, using rat liver specimens. To precisely compare DNA quality via pair analysis, matched samples of fresh-frozen tissues and FFPE tissues were prepared from a single specimen. DNA quality was assessed by quantitative PCR (qPCR), as previously described [4]. We also clarified an essential step for DNA extraction from formalin-fixed (FF) and FFPE tissues. 2) We sought to ascertain whether DNA extracted from FFPE sections after IHC staining can be used. FFPE thin sections are subjected to complex processing during IHC staining, and it is unknown how and whether the quality and quantity of DNA are altered and whether FFPE DNA after IHC can be applied for genetic analysis. To address these concerns, we determined both DNA quality and quantity at each step of IHC. 3) We also assessed whether artifactual mutations occur during FFPE tissue preparation. It has been reported that such artifactual mutations are caused by FFPE preparation [5–7], whereas other studies report that this does not occur [8–10]. To resolve this disagreement, we compared a pair of fresh-frozen and FFPE specimens from the same tissue by comprehensive genetic sequencing. Although there have been a few reports on such investigations of cancerous tissues with somatic mutation [6–10], mutation-positive cancerous tissues are troublesome due to intra-tumor heterogeneity [1, 11, 12], which can induce sampling error. In this study, we extracted DNA from 4 pairs of fresh-frozen and FFPE tissues of a single normal liver with no mutation and compared NGS results in each case by pair analysis.

## Materials and methods

### Human tissue specimens

We obtained 10 normal and 7 tumorous liver tissues by surgical resection of liver metastasis of colorectal cancer and hepatocellular carcinoma, respectively. Four normal breast tissues and 6 tongue tumor tissues were obtained by surgical resection of primary breast cancer and tongue cancer, respectively. All 27 samples were routinely formalin fixed and paraffin embedded in the pathological diagnostic departments of our hospitals: 14 tissue samples were fixed with 10% formalin, 7 tissue samples were fixed with 20% formalin, and 6 tongue tumor tissue samples were fixed with 10% neutral buffered formalin (10% formalin, 29mM NaH_2_PO_4_, 46mM Na_2_HPO_4_). Ten samples of normal liver (h16 to h25) paired with FFPE samples were separately stored at -80°C. Our study protocol was previously approved by the ethics committees of Nihon University School of Medicine and Nihon University School of Dentistry at Matsudo, in accord with the 1975 Declaration of Helsinki. All patients provided written informed consent.

### Rat liver specimens

Rat liver tissues were obtained from 6 male Sprague Dawley rats at ages of 17 to 37 weeks. To prepare 3 matched specimens, i.e., fresh-frozen, FF and FFPE tissues from a single specimen, liver tissues from 3 rats (r1 to r3) were individually divided into 2 pieces. One piece was stored at -80°C, and the other piece was fixed with 10% neutral buffered formalin. One day after fixation, the fixed tissue was divided into 4 pieces, of which 3 pieces were fixed for additional 1, 2, or 3 days. After fixation, the tissues were washed with 2.5 L of distilled water 4 times for 10 minutes each. Half of the sample was stored at -80°C (r1FF, r2FF, r3FF), and the other half was paraffin embedded (r1FFPE, r2FFPE, r3FFPE) (S1 Fig). To prepare another 3 specimens for 5-day fixation, paired fresh-frozen and FFPE tissues were prepared from liver tissues of 3 rats (r4 to r6). These three FFPE tissues were stored at room temperature for 6 months, and the sections were stored at -80°C for one month (S1 Fig). Animal experiments were conducted with approval from the Nihon University Animal Care and Use Committee and in accordance with the institutional animal care guidelines of Nihon University.

### Immunohistochemical staining

Four FFPE tissues (h16 to h19) were sliced into thin sections of 10 μm in thickness (a). Ten micrometer-thick sections on microscope slides were deparaffinized (b), and the sections were then treated with 0.01 M citrate buffer, pH 6.0 at 120°C for 15 minutes for antigen retrieval (c). After inactivation of endogenous peroxidase with 1% hydrogen peroxide in methanol (d), the sections were blocked with 5% skim milk in phosphate-buffer saline (PBS) for 30 minutes at 37°C (e). The sections were incubated with a 1/200 dilution of mouse anti-human hepatocyte monoclonal antibody (Clone OCH1E5) (Dako, Glostrup, Denmark) at 37°C for 1 hour and then with Histofine Simple Stain MAX PO (MULTI) (Nichirei, Tokyo, Japan) for 30 minutes at room temperature (f). The sections were colorized with the chromogen 3,3'-diaminobenzidine (Cat. No. 40651) (MUTO PURE CHEMICALS, Tokyo, Japan) (g). The thin section was washed with distilled water after each of the 6 steps ((b) to (g)); the section was then stripped from the slide and stored at -80°C until use.

### DNA extraction

DNA was extracted from frozen and FF tissues using the phenol-chloroform method, as described previously [4]. DNA extraction from FFPE samples was performed using the RecoverAll Total Nucleic Acid Isolation kit (Life Technologies, Carlsbad, CA, USA) according to the manufacturer’s protocol. Protease digestion in both procedures was performed overnight at 55°C with gentle rotation. Heat treatment at 95°C for 30 minutes was included or omitted after digestion to validate the heat treatment. All DNA samples were purified by ethanol precipitation and dissolved in distilled water.

### Quality assessment of DNA

Samples of 400 ng rat DNA and 500 ng human DNA were subjected to 0.8% agarose gel electrophoresis. DNA on a gel were stained with GelRed (Biotium, Inc., Fremont, CA, USA). Samples of 10 ng DNA were subjected to polymerase chain reaction (PCR) to amplify 301 bp and 952 bp of the rat Tp53 gene in a 20-μL reaction containing TaKaRa Ex Taq Hot Start Version (Takara, Shiga, Japan) as follows: preheating at 94°C for 60 seconds, 35 cycles of 98°C for 10 seconds, 60°C for 30 seconds and 72°C for 60 seconds, and a final extension at 72°C for 5 minutes. A sample of the 10-μL reaction was subjected to 2% agarose gel electrophoresis. The primer sequences used for PCR are shown in S1 Table.

Quantitative PCR (qPCR) of human (93 bp) and rat (174 bp) *glyceraldehyde-3-phosphate dehydrogenase* (*GAPDH*) was performed as described previously [4] using TaqMan (Life Technologies) (S1 Table). The ratio of sample DNA quality to frozen tissue DNA was calculated by the ΔCt method, and the results are expressed as 2^-ΔCt^.

### Amplicon sequencing

Four pairs of frozen tissue and FFPE DNA samples from normal liver (h16, h17, h18, h19) were subjected to amplicon sequencing using Ion AmpliSeq Comprehensive Cancer Panel (Life Technologies) according to the manufacturers’ protocols, with some modifications. This panel consists of approximately 16,000 primer pairs covering all exons of 409 cancer-associated genes (1.6 megabases of target sequence). Eighty nanograms of DNA from 8 samples were used to prepare barcoded libraries with IonXpress barcoded adapters. Sequencing was performed using an Ion Proton Sequencer (Life Technologies) with the Ion PI chip. All data were analyzed with Torrent Suite. Candidates for FFPE-specific single-nucleotide variants (SNVs) were identified by Tumor-Normal pair analysis version 5.0 of Ion Reporter using paired FFPE DNA and frozen tissue DNA as the tumor and normal samples, respectively.

### Sanger sequencing

Mutations in *FGFR3*, *MAGI1* and *CHEK2* were examined by Sanger sequencing. PCR amplification of these 3 regions was performed using TaKaRa Ex Taq Hot Start Version (Takara) and the primer pairs shown in S1 Table. The reaction was as follows: after preheating at 94°C for 1 minute, 35 cycles of 98°C for 10 seconds and 60°C or 65°C for 1 minutes, and a final extension at 72°C for 5 minutes. The PCR products were purified using the illustra ExoProStar 1-Step kit (GE Healthcare UK Ltd, Little Chalfont, UK) and then sequenced using the BigDye Terminator version 1.1 cycle sequencing kit (Life Technologies). After purification using the BigDye Xterminator kit (Life Technologies), the nucleotide sequences were determined using an ABI310 Genetic Analyzer (Life Technologies).

### SYBR green allele-specific qPCR

Allele-specific qPCR was quantitatively performed using the THUNDERBIRD SYBR qPCR Mix (TOYOBO, Osaka, Japan) with a StepOnePlus (Life Technologies). Two allele-specific primers for wild-type and mutant sequences and a single opposite-directed primer were designed by adjusting their melting temperatures to a similar temperature between 60°C and 66°C, as based on the Nearest Neighbor’s method for melting temperature calculation [13] (S1 Table). PCR was performed in a 10-μL reaction by preheating at 95°C for 60 seconds, followed by 40 cycles of 95°C for 15 seconds and 60°C to 66°C for 60 seconds. A ratio of mutant allele to wild-type allele was calculated as 2^-ΔCt^, in which ΔCt is the result of subtracting the Ct value of the wild-type PCR from that of the mutant PCR. The mutant-allele frequency was calculated as 2^-ΔCt^/ (1+2^-ΔCt^).

### TaqMan mutation detection assay (MDA)

*KRAS* mutations were estimated using TaqMan MDA (Life Technologies); mutations at codon 12 (c.34G>A, c.35G>A) and 13 (c.38G>A) were assessed by individual assay kits, as shown in S1 Table, according to the manufacturer’s protocol. A *KRAS* reference assay was used to measure the *KRAS* gene quantity. The mutant-allele frequency was determined by 2^-ΔCt^, in which ΔCt is the result of subtracting the Ct value of the reference PCR from that of the mutant PCR.

### Statistical analysis

Statistical analyses to compare formalin concentrations in clinical diagnostic samples were performed using the Mann-Whitney *U* test. The paired *t*-test was used to compare heat treatment of the lysate and the 6 steps of the immunostaining procedure. A p value less than 0.05 was considered significant.

## Results

### DNA quality of FFPE tissues according to formalin fixation

**Fig 1:**
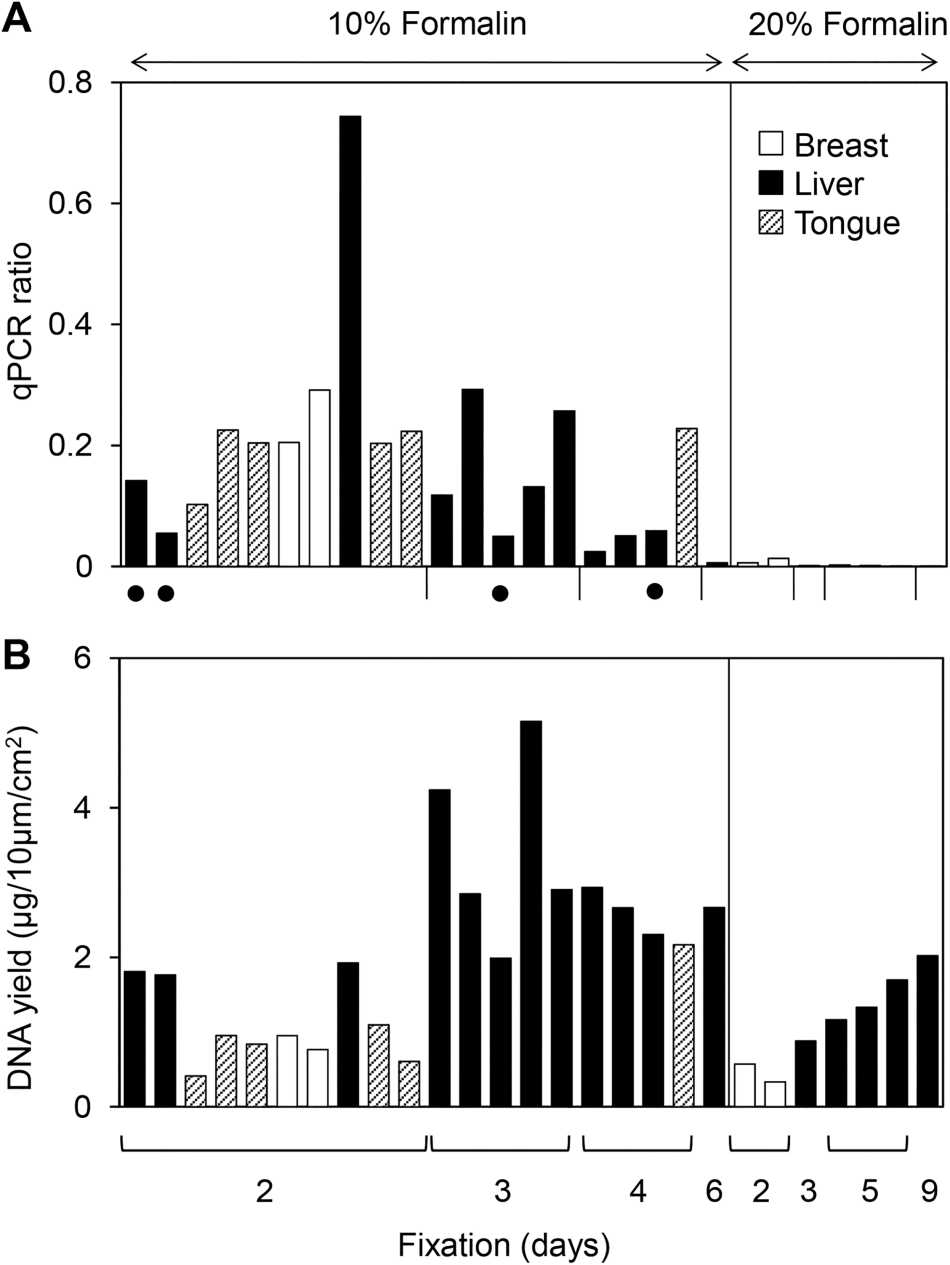
DNA quality and quantity of various clinical diagnostic FFPE tissues. DNA was extracted from 27 FFPE samples of normal breast, normal liver and tumorous tongue and then qualified and quantified. The samples were divided into two groups to test the formalin concentration and subdivided for the number of fixation days. Six tongue tissues were fixed with 10% neutral buffered formalin. The four samples with black dots were fixed at a temperature higher than 25°C. (A) The DNA quality was determined by qPCR of human *GAPDH* (93 bp), and the results are expressed as the ratio to fresh-frozen DNA. (B) The DNA yield was determined using an UV spectrophotometer, and the results are expressed as DNA quantity per cm^2^ of tissue area with 10 μm thickness.

We examined the DNA quality of FFPE specimens routinely used in the hospital department of diagnostic pathology. We determined DNA quality using a relative qPCR ratio of FFPE tissue DNA to fresh-frozen tissue DNA and compared between FFPE samples fixed with 10% formalin and 20% formalin, also considering various fixation time periods (Fig 1A). The DNA quality of 10% formalin-fixed samples was reduced to 0.18±0.16 (average ± standard deviation), and that of 20% formalin-fixed samples was markedly deteriorated to 0.003±0.004. When using 10% formalin, the DNA quality was decreased by a fixation period of longer than 3 days (p<0.01 by the Mann-Whitney *U* test) (Fig 1A); the two-group comparison was performed using 11 cases (2 and 3 days) and 4 cases (4, 5, and 6 days) except 4 dotted cases and one outlier (qPCR = 0.74). An increase in the ambient temperature during fixation also decreased DNA quality (dot samples in Fig 1). However, the quantity of DNA extracted from the FFPE samples was similar among all samples (Fig 1B).

**Fig 2:**
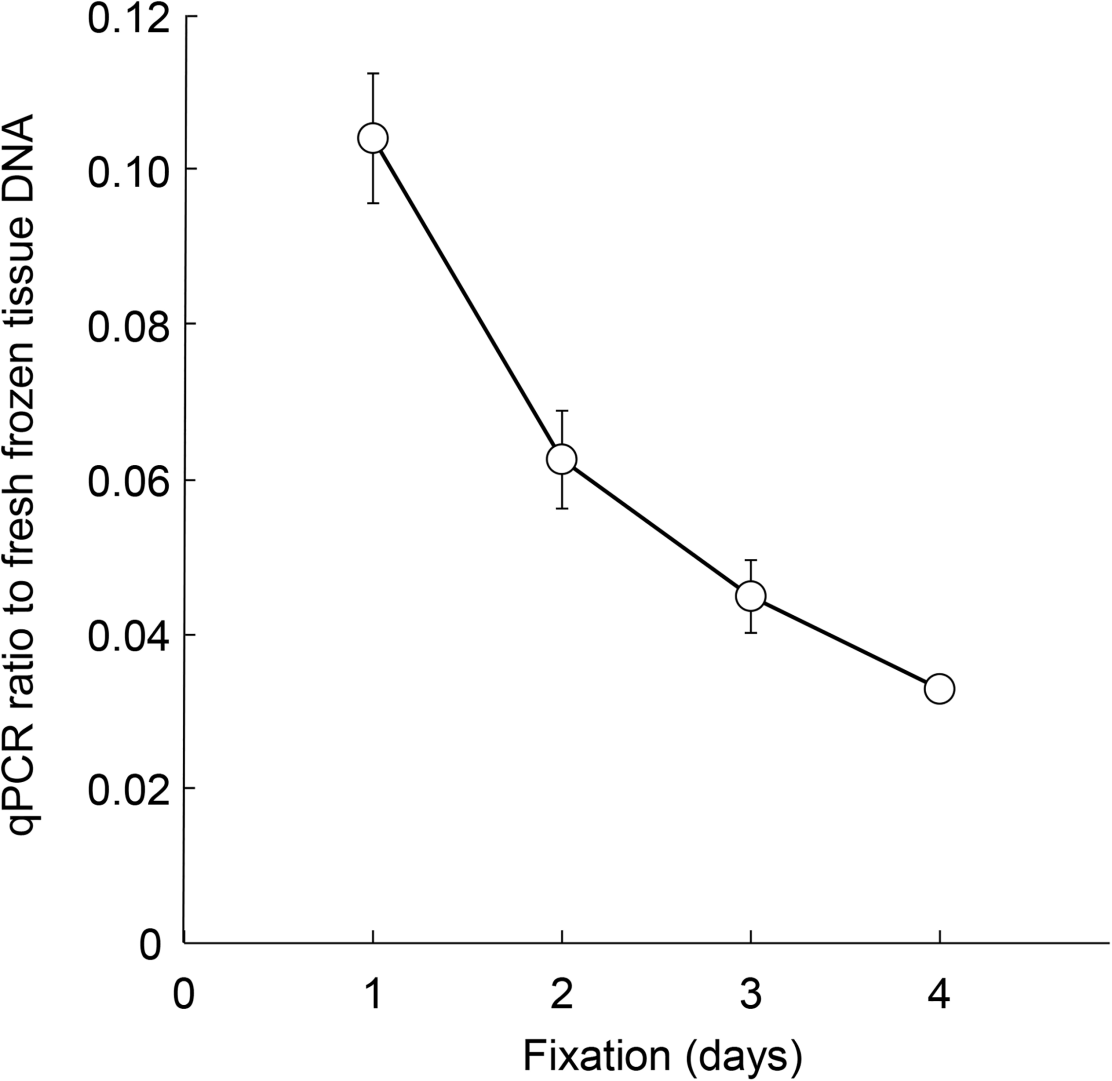
FFPE DNA quality is dependent on the formalin fixation duration. Three sets of 5 matched samples containing fresh-frozen tissue DNA and 4 FFPE (1-, 2-, 3- and 4-day fixation) DNA samples were prepared from 3 rat liver specimens (r1, r2, r3) and examined for DNA quality. The data are shown as averages (circles) with standard deviations (bars).

**Fig 3:**
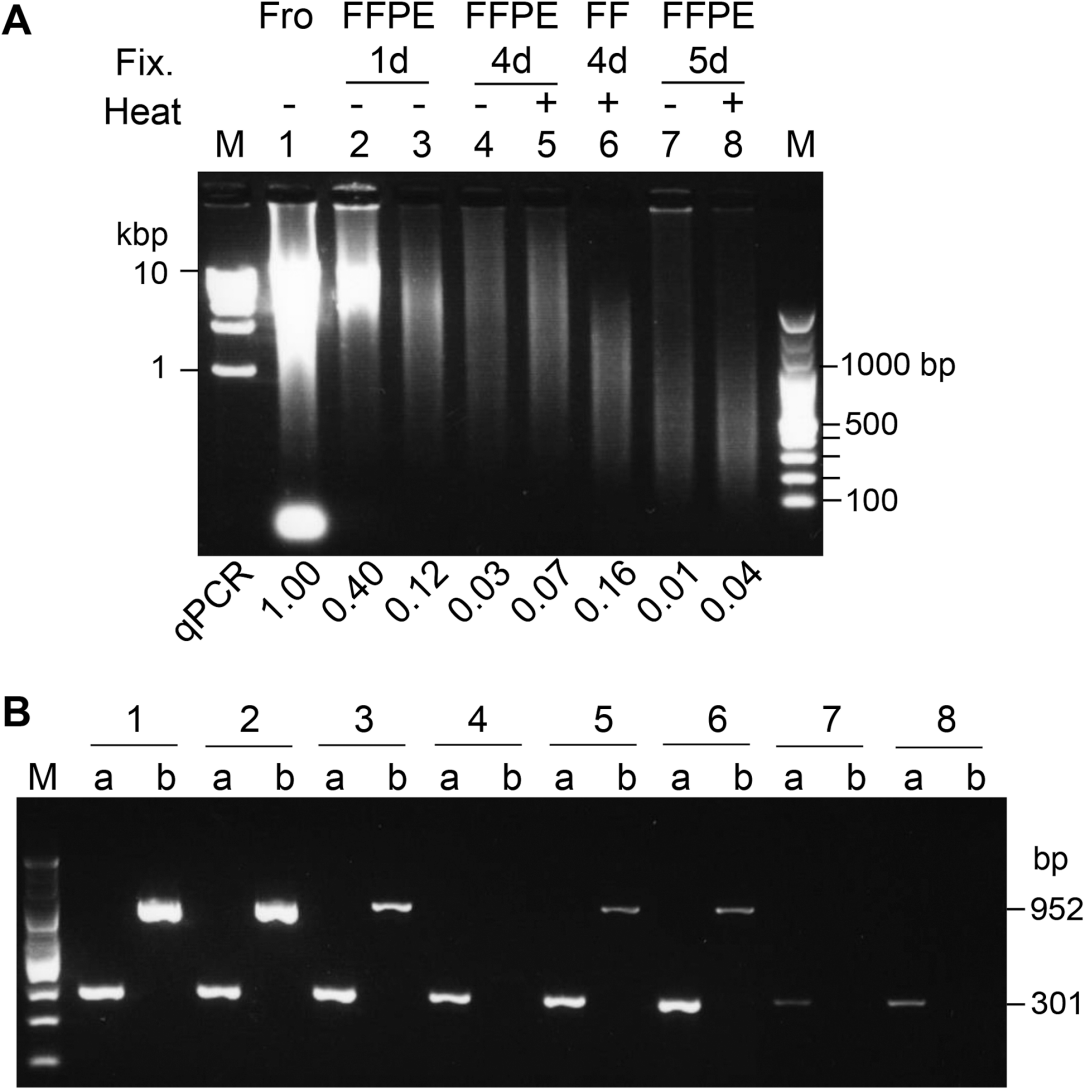
Agarose gel electrophoresis of rat liver DNA. (A) Agarose gel electrophoresis (0.8%) of 400 ng of purified DNA. DNA on the gel were stained with GelRed which is sensitive to double-stranded DNA. The qPCR ratio to matched frozen tissue DNA is shown at the bottom of each lane. Fro, frozen tissue DNA; Fix., fixation for 1 day (1 d): lanes 2 and 3 were different tissue samples with 1 day fixation, 4 days (4 d) and 5 days (5 d); Heat, heat treatment of lysate (+) or not (-) at 95°C for 30 minutes; M, molecular weight marker; qPCR, qPCR ratio to frozen tissue DNA. (B) Agarose gel electrophoresis (2%) of PCR products with target sizes of 301 bp (a) and 952 bp (b). The rat *Tp53* gene was amplified from the indicated DNA, lanes 1 to 8 in (A).

Next, to precisely examine the effect of formalin fixation periods on DNA quality, we prepared 3 sets of 5 matched rat liver samples: fresh-frozen tissue DNA and 4 FFPE DNA samples from the same rat liver fixed with 10% neutral buffered formalin for various periods (S1 Fig). DNA quality significantly and gradually worsened when formalin fixation was performed for longer than 1 day (Fig 2). Agarose gel electrophoresis of FFPE DNA revealed smaller DNA smear sizes from 4-day (lane 4) and 5-day (lane 7) fixed samples compared to 1-day (lane 3) fixation (Fig 3A). The amounts of PCR products with longer size were also decreased in 4 day (lane 4b) and 5 day (lane 7b) compared to the 1 day fixed sample (lane 3b) (Fig 3B).

### DNA quality is enhanced by heat treatment of lysates from FFPE tissues

**Fig 4:**
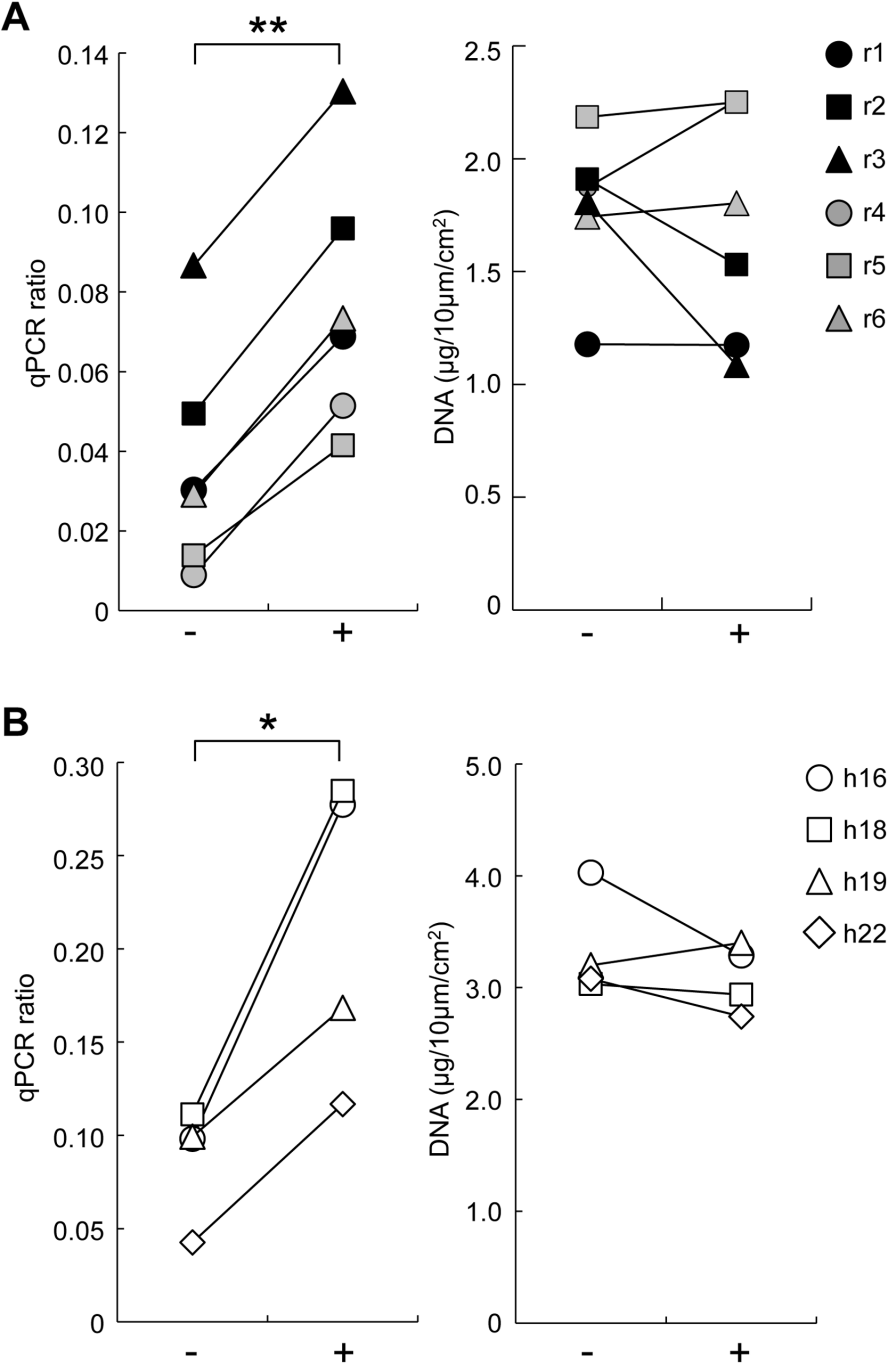
Effects of heat treatment of FFPE lysates on DNA quality and quantity. (A) Rat liver FFPE specimens: 6 matched frozen and FFPE tissues (r1 to r3 fixed for 4 days and r4 to r6 fixed for 5 days shown in S1 Fig) were used, and the FFPE tissue lysates were treated (+) or not (-) at 95°C for 30 minutes before purification. DNA quality and quantity were determined as shown in Fig 1. (B) Human normal liver FFPE specimens: 4 matched frozen and FFPE tissues (h16, h18, h19 and h22) were used, and the FFPE tissue lysates were heat treated and examined for DNA quality (left) and quantity (right), as described in (A). **, p<0.01; *, p<0.05 by the paired *t*-test.

FFPE tissue lysates are occasionally treated with high temperatures to inactivate proteases; however, the manual for the FFPE DNA extraction kit used in the present study does not include this step. To examine the effect of heat treatment on DNA quality and quantity, we prepared a pair of FFPE and fresh-frozen tissues from 6 rat livers (r1 to r6 in S1 Fig). After protease digestion, the FFPE tissue lysates were treated or not at 95°C for 30 minutes and then purified. The DNA quality according to the qPCR ratio was increased in all samples treated with heat, with an average fold increase of 2.8±1.4 (Fig 4A). In contrast, the DNA yield did not change significantly (Fig 4A). Human liver FFPE tissues (h16, h18, h19, h22) were also examined for heat treatment, and the qPCR ratio was determined using paired fresh-frozen tissue DNA. The quality of DNA was similarly improved by heat treatment, by 2.4(±0.4)-fold, though there was no change in DNA yield (Fig 4B).

Agarose gel electrophoresis indicated that the smear size was not altered but that the intensity of double-stranded DNA was increased by heat treatment (lanes 4 vs 5, lanes 7 vs 8 in Fig 3A). The amounts of PCR products with longer size were also increased in 4 day and 5 day with heat treatment (lanes 4b vs 5b, and lanes 7b vs 8b in Fig 3B).

### Heat treatment affects DNA extraction from FF tissues

**Fig 5:**
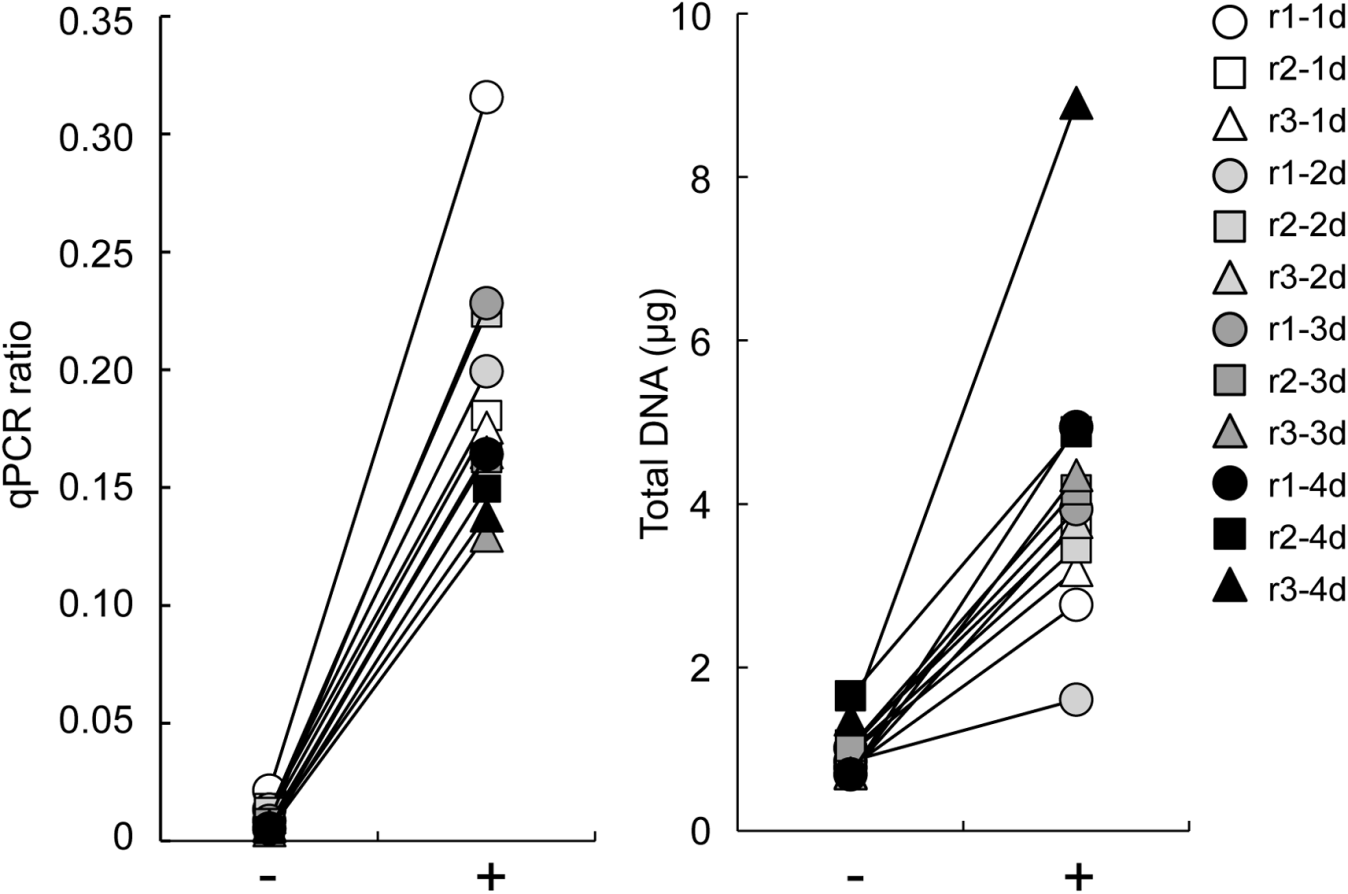
FF DNA quality and quantity dependent on the heat treatment. Three sets of 5 matched samples containing fresh-frozen and FF (1-, 2-, 3- and 4-day fixation) tissues were prepared from 3 rat liver specimens (r1, r2, r3). FF DNA was extracted from the lysates with (+) or without (-) heat treatment and examined for DNA quality (left) and DNA quantity (right).

To examine only the effects of formalin fixation on DNA quality and quantity, we prepared 3 sets of 5 matched samples of fresh-frozen, FF (for 1, 2, 3 and 4 days) tissues from 3 rat livers (r1, r2, r3 in S1 Fig). The qPCR ratio of FF DNA was only 0.015±0.005 for the fixation period of 1 day, whereas that of the FF DNA extracted from the heat-treated lysate was notably increased to 0.224±0.065 (The left in Fig 5). qPCR quality was not influenced by the fixation period. Conversely, the DNA yield was markedly decreased by FF, whereas heat treatment at 95°C for 30 minutes after lysis increased the DNA yield by 4.3±1.5-fold (The right in Fig 5). When comparing the qPCR quality of matched FF and FFPE DNA with heat treatment, FFPE DNA still showed a slight decrease in quality (FF, 0.15±0.01 from r1-4d, r2-4d, and r3-4d in Fig 5; FFPE, 0.098±0.025 from r1, r2, and r3 in Fig 4A). Therefore, formalin fixation is a major cause of reduced quality of DNA from FFPE tissues, and heat treatment after lysis can dramatically improve the quality. Therefore, we applied heat treatment to all lysates of FF and FFPE tissues prior to DNA purification.

### FFPE DNA quality and quantity are altered by the IHC procedure

**Fig 6:**
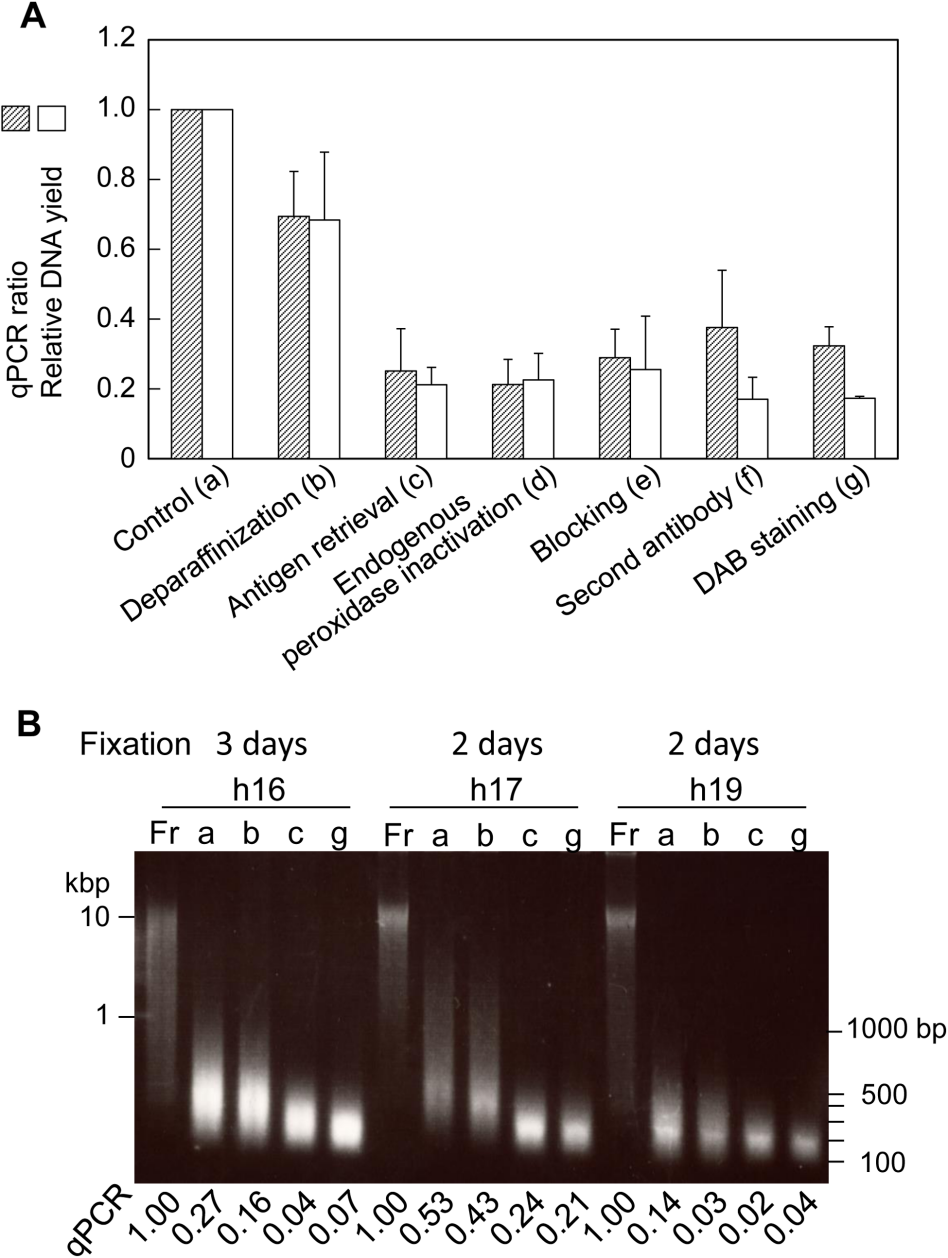
DNA quality and quantity during the course of IHC staining of FFPE thin sections. (A) The DNA qPCR ratio and relative DNA yield were expressed using FFPE thin sections before the start of IHC used as the control. The IHC procedure was divided into 6 steps. Four FFPE samples (h16 to h19) were used for human hepatocyte marker immunostaining. The qPCR value and DNA yield relative to the matched control were calculated, and the mean values of 4 samples were compared among the 6 steps. Control DNA was prepared directly from matched FFPE thin sections according to our modified RecoverAll protocol with additional heat treatment. (B) Agarose gel (0.8%) electrophoresis of 3 representative sets (h16, h17 and h19) of matched DNA samples (500 ng/lane). Fr, frozen tissue DNA; a, b, c, g, DNAs as shown in (A).

To determine whether FFPE DNA from IHC-positive cells can be applied to genome analysis, we measured DNA quality at 6 steps during the immunohistochemistry procedure. Both the quality and quantity of DNA were decreased after antigen retrieval with 0.01 M citrate buffer, pH 6.0, at 120°C for 15 minutes (Fig 6A). Agarose gel electrophoresis showed that the DNA was more severely fragmented with than without antigen retrieval (Fig 6B). However, the qPCR ratio with antigen retrieval was one-fourth that of the starting FFPE material used as a control (Fig 6A). The final qPCR ratio for frozen tissue DNA was at least 0.02 after IHC staining (Fig 6B).

### Comparison of NGS of FFPE DNA with that of paired fresh-frozen tissue DNA

**Fig 7:**
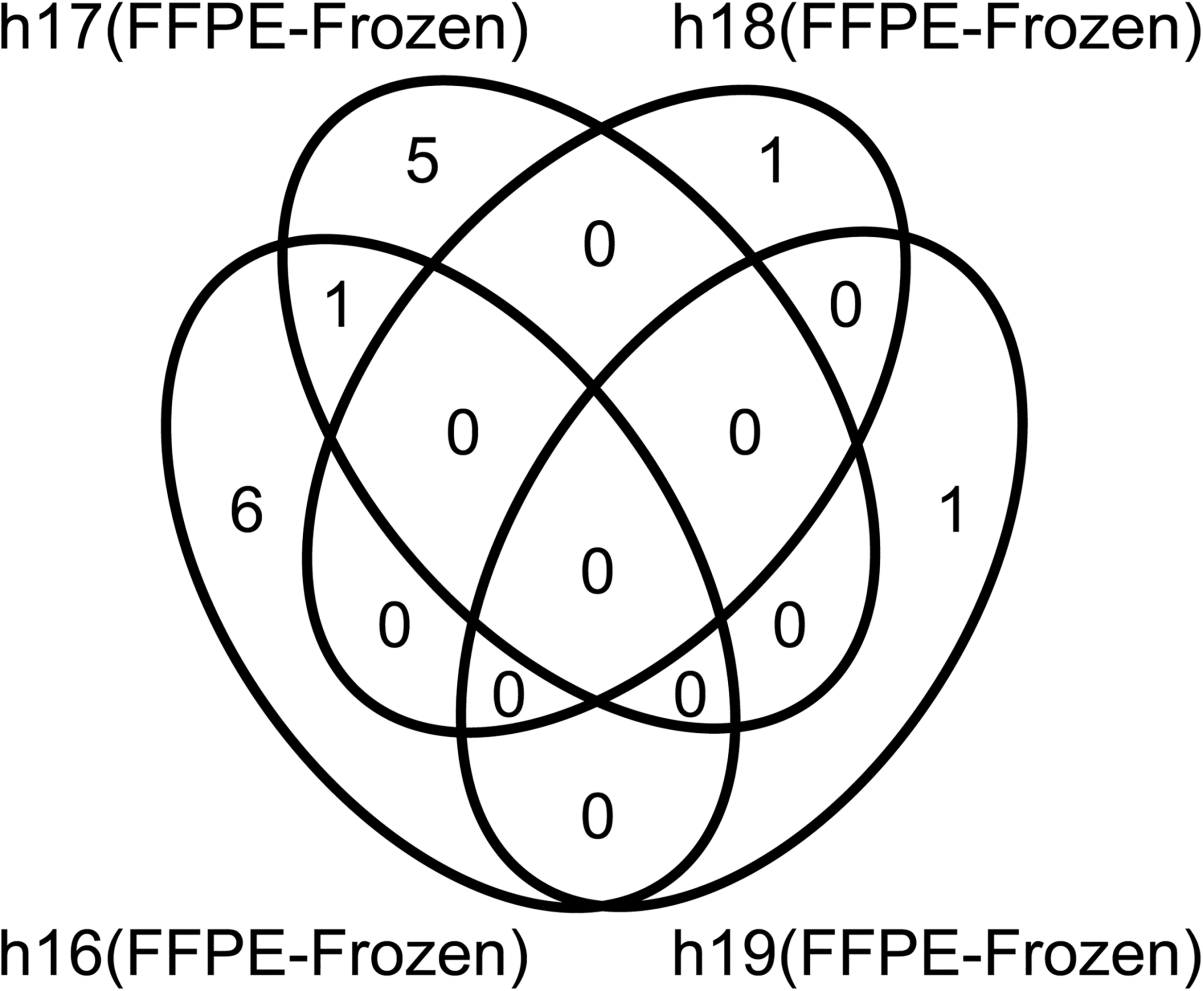
Venn diagram of variant candidates found in 4-pair analyses of FFPE and frozen tissue DNA. Variants identified in 4 matched FFPE-frozen pairs of normal liver tissues, h16, h17, h18 and h19. Fourteen variants were identified by pair analysis using Ion Reporter version 5.0 with the following filters: coverage more than 100, allele frequency more than 0.05, frozen allele coverage = 0. *ERCC2* was a common variant among two samples, h16 and h17.

**Fig 8:**
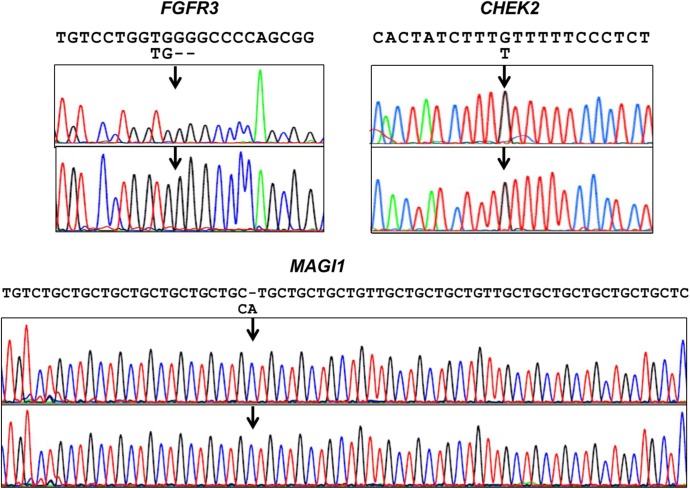
Sanger sequencing of *FGFR3*, *CHEK2*, and *MAGI1*. Variant calls at high frequency were invalidated by Sanger sequencing of amplified products from matched fresh-frozen DNA (upper panel) and FFPE DNA (lower panel). Arrows indicate the positions of a deletion (*FGFR3)*, an SNV (*CHEK2)* and an insertion (*MAGI1*), and the variant sequences are shown below the reference sequences.

FFPE DNA has recently been used for NGS. However, there is still concern that formalin-induced modification and oxidation during storage result in nucleotide sequence artifacts [5–7]. To examine FFPE-dependent artifactual mutation, we compared FFPE DNA (without heat treatment) with frozen tissue DNA from the same samples: 4 pairs of DNA samples were prepared from 4 normal human liver tissues without mutation, and amplicon sequences of 409 cancer-related genes were compared. The NGS data were compatible between the paired DNA samples as shown in Table 1. Nineteen candidates for artifactual mutations that would have been introduced during the FFPE preparation were identified (Fig 7 and Table 2). Of these, nine were examined by Sanger sequencing (Fig 8) or by more sensitive allele-specific qPCR (Table 3). However, none were reproducible, and therefore, artifactual mutations were not present in the FFPE samples used in this study. That means that the potential mutations identified by NGS were probably errors resulting from the NGS, and were not the result of the FFPE preparation.

**Table 1:** Coverage depth of amplicon sequencing of the comprehensive Cancer Panel.

Sample		Mapped reads	% Reads on target	Average base coverage depth
Type	No.			
Frozen	16	10,334,156	97.84	655
	17	8,490,826	98.86	548
	18	9,423,931	97.89	600
	19	10,014,736	98.94	648
FFPE	16	8,489,832	99.47	535
	17	18,169,101	99.52	1179
	18	10,002,318	99.46	646
	19	7,413,303	98.91	469

**Table 2:** Nineteen variant candidates.

Chromosome#: position	Gene	Variant	Sample No.	FFPE			Frozen		Reference sequence ^a^
				Coverage		Allele frequency	Coverage		
				Total	Allele		Total	Allele	
Chr11:3723808	*NUP98*	TGGGGCC>TGGGCCA	17	882	882	1.000	394	0	TCCAG[TTGGGGCCCC]AACCA
Chr4:1801137	*FGFR3*	TGGG>TG	17	369	369	1.000 *	74	0	TGTCCTGG[TGGGG]CCCCAGC
Chr22:29108019	*CHEK2*	C>A	17	1115	1085	0.973 *	1402	0	AGAGGG[AAAAACAAA]GATAG
Chr3:65425582	*MAGI1*	C>CA	16	206	196	0.951 *	185	0	[TGCTGCTGCTGCTGCTGCTG]
Chr12:49445171	*KMT2D*	A>AG	17	285	44	0.154	203	0	GTGGCTCCTCAGCCTGCGGA
Chr19:45864930	*ERCC2*	G>C	17	662	85	0.128 **	71	0	CGGGGTC[GGGGGG]CAGACGG
			16	596	58	0.097 **	53	0	
Chr1:11298662	*MTOR*	G>A	19	128	14	0.109 **	428	0	GCGAAC[AAATTGGG]TCAGAG
Chr3:89528672	*EPHA3*	T>C	16	403	41	0.102 **	178	0	CGGAAGTG[CTTC]TGGACGGA
Chr7:2972136	*CARD11*	A>T	17	177	14	0.079 **	23	0	GACAGATGAGACAGGCCAAG
Chr2:223158895	*PAX3*	T>C	16	117	8	0.068	69	0	CTTACCTCGCTCGCTCAGGA
Chr1:220808673	*MARK1*	T>C	18	172	11	0.064	87	0	C[TTTTTTTTTTTTTTTTTT]G
Chr5:180056483	*FLT4*	C>A	16	230	14	0.061 **	78	0	CACAGCTA[CCCC]ACCGAAGG
Chr16:89877535	*FANCA*	A>C	16	396	22	0.056	740	0	CAAAATCT[AAAA]CCAAAGAC
Chr9:136918526	*BRD3*	TC>GT	16	267	14	0.052	58	0	TTGGAGACCTCCGGGGGGGG
Chr7:55238239	*EGFR*	C>A	16	272	11	0.040	699	0	TGCCACTGAGCCTCATGCCT
Chr1:162737153	*DDR2*	A>T	19	286	10	0.035	796	0	GGAGAAGGTGAGGAGGTGCA
Chr3:65439047	*MAGI1*	T>A	17	299	10	0.033	220	0	AAGAAATTTGTTTGAAAAAA
Chr11:71725198	*NUMA1*	T>TC	17	689	21	0.030	503	0	TGGGGCCTGTTGGCTCTGTC
ChrX:70612697	*BCYRN1*	G>T	18	432	13	0.030 **	824	0	AGGTTTTTAGGAGTCTCACT

A pair of FFPE and frozen tissue DNA samples were subjected to Tumor-Normal Pair Analysis (version 5.0) in Ion Reporter. Fourteen variants were extracted by filtering, as follows: coverage>100, allele frequency>0.05, frozen allele coverage = 0. Five others were extracted by additional filtering of allele frequency>0.03 and frozen coverage>FFPE coverage x 0.8.

^a^ Reference sequences are shown; underline, variant position; square brackets, repeated sequences surrounding the variant position.

*, Variations were analyzed by Sanger sequencing

**, Variations were analyzed by allele-specific qPCR.

**Table 3:** Invalidation of the variant candidates by allele-specific qPCR.

Gene	Variant	Sample No.	FFPE			Frozen		
			Allele frequency			Allele frequency		
			NGS	(coverage)	qPCR	NGS	(coverage)	qPCR
*ERCC2*	G>C	16	0.097	(596)	**0.000**	0	(53)	**0.000**
		17	0.128	(662)	**0.000**	0	(71)	**0.000**
		18	0.287	(508)		0	(112)	
		19	0.183	(485)		0.147	(354)	
*CARD11*	A>T	16	0.192	(73)	**0.000**	0	(48)	**0.000**
		17	0.079	(177)	**0.000**	0	(23)	**0.000**
		18	0.096	(83)		0.053	(57)	
		19	0.098	(102)		0.007	(141)	
*EPHA3*	T>C	16	0.102	(403)	**0.000**	0	(178)	**0.001**
		17	0.033	(809)	**0.001**	0	(105)	**0.000**
		18	0.032	(498)		0.013	(153)	
		19	0.025	(321)		0.026	(383)	
*MTOR*	G>A	16	0.106	(66)		0	(345)	
		17	0.054	(607)		0	(166)	
		18	0.073	(342)		0.009	(223)	
		19	0.109	(128)	**0.006**	0	(428)	**0.009**
*FLT4*	C>A	16	0.061	(230)	**0.007**	0	(78)	**0.012**
		17	0.036	(787)	**0.005**	0	(157)	**0.013**
		18	0.047	(471)		0.015	(198)	
		19	0.040	(276)		0	(428)	
*BCYRN1*	G>T	16	0	(422)		0	(201)	
		17	0.012	(591)		0	(210)	
		18	0.030	(432)	**0.003**	0	(824)	**0.003**
		19	0.016	(122)		0.006	(309)	

The allele frequency and coverage were determined using Ion Torrent Suite, as shown in Table 2.

## Discussion

We here demonstrate three novel points with regard to the utility of FFPE DNA. 1) FFPE DNA of the best quality is obtained by tissue fixation with 10% neutral buffered formalin for 1 day and heat treatment of tissue lysates at 95°C for 30 minutes. 2) IHC staining of FFPE decreases FFPE DNA quantity and quality to one-fourth; antigen retrieval (at 120°C for 15 minutes, pH 6.0) is the major cause of these decreases. 3) No artifactual mutation occurs during FFPE preparation of non-mutated tissue specimens, as shown by precise comparison of NGS results from FFPE DNA and frozen tissue DNA extracted from the same normal liver specimen.

FFPE tissues used for morphological examination in routine pathological diagnosis have occasionally been prepared by fixation with 20% formalin because of the high structural quality resulting from this treatment. However, 20% formalin fixation markedly decreased the quality of FFPE DNA to one-sixtieth of the value when using 10% formalin (Fig 1). To determine the precise difference between the two concentrations of formalin, three sets of three matched rat liver DNA samples from 10%- and 20%-neutral buffered formalin-fixed and fresh-frozen tissues were also examined for DNA quality. The results showed that compared with 10% neutral buffered formalin, 20% neutral buffered formalin decreased the DNA quality to one-fourth (data not shown). Therefore, to obtain the best DNA quality, fixation should be performed in 10% neutral buffered formalin for 1 day (Fig 2). However, due to antigen preservation for IHC diagnosis, FFPE tissues are prepared for routine pathological diagnosis by fixation with 10% formalin for 2 to 3 days (Fig 1). Although the quality of DNA from FFPE tissues fixed for 2 days is approximately half that of DNA from tissue fixed for 1 day, the former can be used. In addition, we noticed that the thickness of the tissue is important for formalin penetration: the thinner the tissue, the more quickly the formalin penetrates. In fact, higher DNA quality was also found with thinner tissue (data not shown). The observed occasional variability in DNA quality shown in Fig 1 (0.05 to 0.74) is most likely partly due to differences in tissue size at the formalin fixation step.

Several kits of DNA extraction from FFPE tissues are commercially available, and the manual of most kits includes heat treatment (at 80 to 100°C for 10 to 60 minutes) to inactivate the proteases contained in the lysis buffer. In contrast, the kit used in the present study does not involve high-temperature treatment of the lysate. To our knowledge, this is the first study to determine whether or how treatment at high temperature is important for DNA quality and quantity. Heat treatment increased the DNA quality of FFPE lysates 2- to 3-fold (Fig 4) and of FF lysates 15-fold (Fig 5) and also increased the DNA quantity of FF lysates 4-fold (Fig 5). As FF DNA was extracted using a phenol/chloroform method, the protease in the lysis buffer was sufficiently inactivated by the phenol extraction step; thus, heat treatment possibly activates another function, which results in increased DNA quality and quantity. DNA quality was similar among FF lysates heat treated for 5, 30 and 60 minutes (data not shown). These results suggest the following three mechanisms of heat treatment [3, 14, 15]: 1) breakage of protein-DNA cross-links [16, 17], promoting the transition of DNA from the middle protein phase to the upper aqueous phase and ultimately efficient extraction during the phenol/chloroform step [18]; 2) removal of DNA modifications such as histone-DNA cross-links and formaldehyde-DNA adducts [19, 20]; and 3) breakage of inter-strand DNA cross-links [21], creating effective templates for PCR. Thus, heat treatment at 95°C is essential for DNA extraction from FF and FFPE tissues.

To develop a new era of clinical diagnosis using ‘microgenomics’, it is important to assess whether FFPE DNA after IHC staining can be used for PCR-based genetic analysis. We for the first time evaluated the quality and quantity of DNA from FFPE sections during the course of IHC. The major cause of reductions in both quality and quantity of DNA is a general antigen retrieval step at pH 6.0 and 120°C for 15 minutes, whereas the other steps exert relatively minor effects. This reduction is not large, one-fourth of the starting FFPE section, and PCR-based analysis is still possible. Although the applicability of FFPE DNA after IHC staining to NGS has not yet been proven, our analysis of FFPE DNA suggests that FFPE DNA with a qPCR ratio of more than 0.02 is an appropriate template for NGS based on amplicon sequencing. To further improve the quality and quantity of FFPE DNA after IHC, other conditions of antigen retrieval should be examined in addition to the temperature, time, and pH of heat treatment.

Recently, the possibility of NGS analysis of FFPE DNA was investigated in several reports [5–10]. As in our study, these other authors applied NGS to determine whether any artifactual mutations exist in FFPE DNA by direct comprehensive comparison with matched frozen tissue DNA [6–10]; despite of the presence of FFPE-specific variant calls in FFPE samples, these disappeared by changing various analysis parameters [6–10]. Therefore, variants with high confidence are highly concordant between paired fresh-frozen and FFPE tissue samples [6, 7, 9, 10]. However, in our study, nineteen variants remained even with high confidence (p value < 1.0 x 10^−7^), and disappeared only when these were verified using Sanger sequencing or more sensitive qPCR. Our samples were non-pathogenic normal tissues without mutation, and then artifactual mutations induced by FFPE were directly determined in comparison with paired fresh-frozen tissue samples. On the other hand, samples of other studies were cancerous with some mutations [6, 7, 9, 10], recently revealed intra-tumor heterogeneity [1, 11, 12]; even paired samples from a single tumor could have different mutations, thus sampling error possibly occurs among FFPE and fresh-frozen tissues. Finally, our study directly indicates that FFPE generates no artifactual mutations, and also suggests that true mutation of NGS data must be confirmed by allele-specific qPCR.

Recently, C>T and G>A transitions in FFPE DNA (artifactual mutations) have been validated by uracil-DNA glycosylase (UDG) treatment of FFPE DNA before pre-amplification for sequence analysis; for example, such transitions can be found in mutational hot spots in the *KRAS*, *BRAF* and *EGFR* genes [22, 23]. If UDG-sensitive variants such as C>U deamination are present in FFPE DNA, UDG treatment reduces the variant frequency of C>T and G>A transitions [22, 23]. For example, the frequency of *KRAS* G>A (c.35) mutation in FFPE DNA changed from 2.1% before UDG treatment to 0.7% after treatment [23]. Regardless, uracil lesions are generated not only by spontaneous deamination in FFPE blocks (artifactual mutation) but also by activation-induced cytidine deaminase in cancer (natural mutation). In general, cytidine deaminase is involved in biological diversity, such as reprograming toward pluripotency, immunoglobulin diversification, immune responses to viral infection and cancer mutagenesis [24–26]. When we examined artifactual mutations at the *KRAS* mutational hot spots by NGS and MDA in normal human liver (S2 Table) and by allele-specific qPCR in normal rat liver (S3 Table), we found no changes. We conclude that no artifactual mutation occurs in FFPE tissues, at least as prepared under the conditions of our study.

## Conclusion

The best DNA quality ratio of FFPE to frozen tissue DNA is obtained by fixation with 10% neutral formalin for 1 day and heat treatment of tissue lysates at 95°C for 30 minutes. IHC staining of FFPE decreases FFPE DNA quantity and quality to one-fourth at the antigen retrieval step (at 120°C for 15 minutes, pH 6.0). Such FFPE DNA can be utilized for NGS. No artifactual mutation occurs during FFPE preparation under the conditions specified here and given the condition that apparent mutations detected by NGS must be verified by more sensitive qPCR.

## Supporting information

S1 Fig Matched liver specimens from 6 rats. The rat liver specimens in this study were prepared from 6 rats (r1 to r6). Three sets of fresh-frozen tissue, FF and FFPE DNA samples were prepared from 3 rat livers (r1, r2, r3), and 4 fixation periods were tested (1, 2, 3 and 4 days). To adjust DNA extraction for the same day, storage periods at -80°C for the FF and FFPE tissues are indicated in a table. Another three sets of frozen tissue and FFPE DNA samples were prepared from 3 rat livers (r4, r5, r6) for testing a long period of fixation and storage.
(PDF)

S1 Table Primer sequences and TaqMan IDs used in this study. ^a^ Upper, forward primer; lower, reverse primer.
^b^ The first nucleotide in parentheses is the wild-type allele, and the second is the mutant allele.
^c^ PCR was performed using 2 annealing temperatures appropriate for each sample: 60°C for fresh-frozen tissue DNA and 65°C for FFPE DNA due to better PCR condition for Sanger Sequencing.
^d^ MDA was performed using 2 annealing temperatures: 58°C for an initial 5 cycles and 60°C for the ensuing 45 cycles.
(PDF)

S2 Table Human *KRAS* mutation analyses by amplicon sequencing and MDA. Amplicon sequencing was performed for 4 pairs of FFPE and frozen tissue DNA. MDA was performed for 6 pairs of FFPE and frozen tissue DNA. qPCR was determined by the ratio of mutant PCR to *KRAS* reference PCR, as described in Materials and Methods.
(PDF)

S3 Table Rat *Kras* mutation analysis by allele-specific qPCR. Allele-specific qPCR of 3 mutations of the *Kras* gene was performed using 6 pairs of FFPE and frozen tissue DNA. The allele frequency was calculated as described in Materials and Methods.
(PDF)
