# Supplementary material for: Assessment of the quality of DNA from various formalin-fixed paraffin-embedded (FFPE) tissues and the use of this DNA for next-generation sequencing (NGS) with no artifactual mutation
Source: PLoS One. 2017 May 12;12(5):e0176280. doi: 10.1371/journal.pone.0176280 (PMC5428915; doi:10.1371/journal.pone.0176280)
Supplement: S1 Fig — The rat liver specimens in this study were prepared from 6 rats (r1 to r6). Three sets of fresh-frozen tissue, FF and FFPE DNA samples were prepared from 3 rat livers (r1, r2, r3), and 4 fixation periods were tested (1, 2, 3 and 4 days). To adjust DNA extraction for the same day, storage periods at -80°C for the FF and FFPE tissues are indicated in a table. Another three sets of frozen tissue and FFPE DNA samples were prepared from 3 rat livers (r4, r5, r6) for testing a long period of fixation and storage. (PDF) [file pone.0176280.s001.pdf]

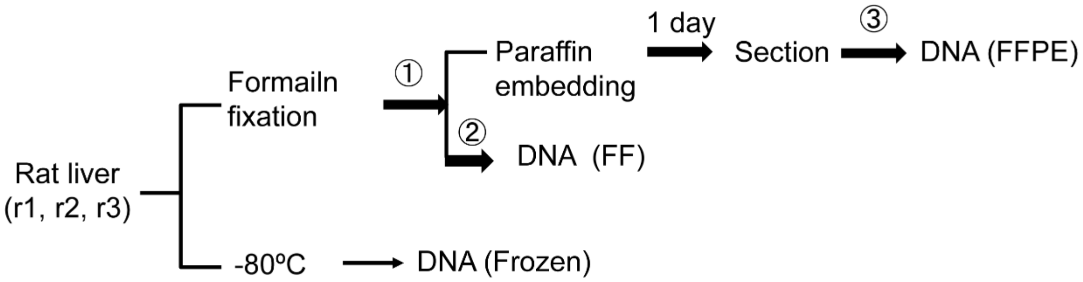

① Fixation	② -80ºC (FF)	③ -80ºC (FFPE)
1 day	4 days	13 days
2 days	3 days	12 days
3 days	2 days	11 days
4 days	1 day	10 days

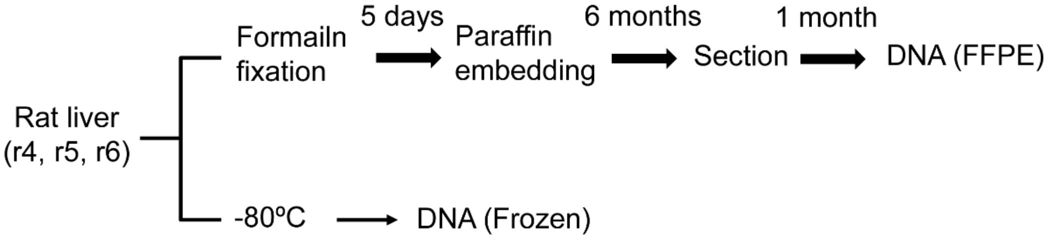

S1 Fig. Matched liver specimens from 6 rats

The rat liver specimens in this study were prepared from 6 rats (r1 to r6). Three sets of fresh-frozen tissue, FF and FFPE DNA samples were prepared from 3 rat livers (r1, r2, r3), and 4 fixation periods were tested (1, 2, 3 and 4 days). To adjust DNA extraction for the same day, storage periods at -80°C for the FF and FFPE tissues are indicated in a table. Another three sets of frozen tissue and FFPE DNA samples were prepared from 3 rat livers (r4, r5, r6) for testing a long period of fixation and storage.
