# Supplementary material for: Assessment of the quality of DNA from various formalin-fixed paraffin-embedded (FFPE) tissues and the use of this DNA for next-generation sequencing (NGS) with no artifactual mutation
Source: PLoS One. 2017 May 12;12(5):e0176280. doi: 10.1371/journal.pone.0176280 (PMC5428915; doi:10.1371/journal.pone.0176280)
Supplement: S1 Table — a Upper, forward primer; lower, reverse primer. b The first nucleotide in parentheses is the wild-type allele, and the second is the mutant allele. c PCR was performed using 2 annealing temperatures appropriate for each sample: 60°C for fresh-frozen tissue DNA and 65°C for FFPE DNA due to better PCR condition for Sanger Sequencing. d MDA was performed using 2 annealing temperatures: 58°C for an initial 5 cycles and 60°C for the ensuing 45 cycles. (PDF) [file pone.0176280.s002.pdf]

S1 Table. Primer sequences and TaqMan IDs used in this study

Experiment	Gene	Primer Sequence (5’-3’) [a]	Annealing (°C)
TaqMan Assay	*GAPDH*	Hs02758991_g1	60
	Gapdh	Rn01775763_g1	60
Fragmentation PCR	*Tp53* 301 bp	CCTCAATAAGCTGTTCTGCC<br>AGTCTGCCTGTCGTCCAGAT	60
	Tp53 952 bp	CCTCAATAAGCTGTTCTGCC<br>GCCTCCACCTTCTTTGTCCT	60
Sanger Sequencing	*CHEK2*	CCCAGGAGTGGTAGGTCTCA<br>GCCTCTCTTGCTGAACCAAT	60/65 [c]
	FGFR3	ACTGTCTGGGTCAAGGATGG<br>AGCTGTAGGCCCCGGAGT	60
	MAGI1	CAGCCACATCAACAGGAAGA<br>CTCAGATTTCACCCCATGCT	60
SYBR Green Allele-specific qPCR	*ERCC2*	GCTCATGCTTCCGTCTG(C/G) [b]<br>TTACCTTTGGCGTAGGTGCT	65
	CARD11	CCAAATCTCTCTTGGCCTG(T/A)<br>GAGGGGACCTTTCAGCAGTT	65
	EPHA3	GGTTGGGCCACAGAAGAAG<br>CCACCACTTCCGTCCAG(A/G)	65
	MTOR	TCTATCCAGGCCACTCTCTGA(C/T)<br>ATGCGGATCTCCTTGTGCT	66
	FLT4	CAAGTGTTCCACCCAAAGAAA<br>CTCCTGCACAGCTACCC(C/A)	65
	FANCD2	TAAAACAAGGAAAGCAAAGTGG(A/G)<br>TATGTCAATCCCCAGAAGCA	65
	BCYRN1 / TAF1	AGAAATGGGTGAGTGGAGGA<br>CCTCAAACAAAAAAAAGTGAGACT(C/A)	65
	*Kras*_ref	TCCACAAAGTGATTCTGAATT<br>CGTAGGATCATATTCATCCACAAA	62

SYBR Green Allele-specific qPCR	*Kras* (c.34)	ACTTGTGGTAGTTGGAGCT(G/A)<br>TCCACAAAGTGATTCTGAATT	62
	Kras (c.35)	CTTGTGGTAGTTGGAGCTG(G/A)<br>CCACCAAGTGATTCTGAATTA	62
	Kras (c.38)	GTGGTAGTTGGAGCTGGTG(G/A)<br>TCCACAAAGTGATTCTGAATT	65
TaqMan Mutation Detection Assay	*KRAS*_ref	Hs00000174_rf	58 (5), 60 (45) [d]
	KRAS (c.34)	Hs00000115_mu	
	KRAS (c.35)	Hs00000121_mu	
	KRAS (c.38)	Hs00000131_mu	

[a] Upper, forward primer; lower, reverse primer.

[b] The first nucleotide in parentheses is the wild-type allele, and the second is the mutant allele.

[c] PCR was performed using 2 annealing temperatures appropriate for each sample: 60°C for fresh-frozen tissue DNA and 65°C for FFPE DNA due to better PCR condition for Sanger Sequencing.

[d] MDA was performed using 2 annealing temperatures: 58°C for an initial 5 cycles and 60°C for the ensuing 45 cycles.
