# Supplementary material for: Assessment of the quality of DNA from various formalin-fixed paraffin-embedded (FFPE) tissues and the use of this DNA for next-generation sequencing (NGS) with no artifactual mutation
Source: PLoS One. 2017 May 12;12(5):e0176280. doi: 10.1371/journal.pone.0176280 (PMC5428915; doi:10.1371/journal.pone.0176280)
Supplement: S2 Table — Amplicon sequencing was performed for 4 pairs of FFPE and frozen tissue DNA. MDA was performed for 6 pairs of FFPE and frozen tissue DNA. qPCR was determined by the ratio of mutant PCR to KRAS reference PCR, as described in Materials and Methods. (PDF) [file pone.0176280.s003.pdf]

S2 Table. Human *KRAS* mutation analyses by amplicon sequencing and MDA

No.	Genotype	FFPE			Frozen		
		Coverage	Allele Coverage	qPCR	Coverage	Allele Coverage	qPCR
16	c.34 G>A	212	0	**6.0E-6**	893	0	**7.7E-7**
	c.35 G>A		0	**1.3E-5**		0	**3.9E-6**
	c.38 G>A		1	**7.4E-7**		0	**8.5E-8**
17	c.34 G>A	1036	0	**1.7E-7**	435	0	**9.7E-8**
	c.35 G>A		2	**8.6E-7**		0	**6.5E-7**
	c.38 G>A		0	**1.2E-7**		0	**2.5E-7**
18	c.34 G>A	272	1	**4.1E-7**	592	0	**1.1E-6**
	c.35 G>A		0	**1.3E-5**		1	**1.3E-5**
	c.38 G>A		0	**5.9E-6**		0	**2.8E-5**
19	c.34 G>A	220	0	**9.7E-7**	572	0	**6.0E-6**
	c.35 G>A		0	**6.1E-6**		0	**4.2E-6**
	c.38 G>A		0	**1.2E-6**		0	**1.7E-6**
20	c.34 G>A			**3.4E-6**			**1.4E-7**
	c.35 G>A			**3.4E-6**			**3.1E-6**
	c.38 G>A			**3.4E-6**			**2.8E-6**
21	c.34 G>A			**2.3E-6**			**1.3E-6**
	c.35 G>A			**9.3E-6**			**1.1E-5**
	c.38 G>A			**2.3E-6**			**1.6E-7**

Amplicon sequencing was performed for 4 pairs of FFPE and frozen tissue DNA. MDA was performed for 6 pairs of FFPE and frozen tissue DNA. qPCR was determined by the ratio of mutant PCR to *KRAS* reference PCR, as described in Materials and Methods.
