# Supplementary material for: Assessment of the quality of DNA from various formalin-fixed paraffin-embedded (FFPE) tissues and the use of this DNA for next-generation sequencing (NGS) with no artifactual mutation
Source: PLoS One. 2017 May 12;12(5):e0176280. doi: 10.1371/journal.pone.0176280 (PMC5428915; doi:10.1371/journal.pone.0176280)
Supplement: S3 Table — Allele-specific qPCR of 3 mutations of the Kras gene was performed using 6 pairs of FFPE and frozen tissue DNA. The allele frequency was calculated as described in Materials and Methods. (PDF) [file pone.0176280.s004.pdf]

S3 Table. Rat *Kras* mutation analysis by allele-specific qPCR

No.	Genotype		FFPE	Frozen
			Allele Frequency	Allele Frequency
r1	c.34	G>A	0.0046	0.0036
	c.35	G>A	0.0116	0.0082
	c.38	G>A	0.0094	0.0151
r2	c.34	G>A	0.0053	0.0066
	c.35	G>A	0.0113	0.0095
	c.38	G>A	0.0148	0.0157
r3	c.34	G>A	0.0029	0.0068
	c.35	G>A	0.0108	0.0087
	c.38	G>A	0.0123	0.0158
r4	c.34	G>A	0.0035	0.0034
	c.35	G>A	0.0097	0.0128
	c.38	G>A	0.0078	0.0969
r5	c.34	G>A	0.0043	0.0028
	c.35	G>A	0.0086	0.0107
	c.38	G>A	0.0167	0.0119
r6	c.34	G>A	0.0038	0.0047
	c.35	G>A	0.0071	0.0098
	c.38	G>A	0.0192	0.0111

Allele-specific qPCR of 3 mutations of the *Kras* gene was performed using 6 pairs of FFPE and frozen tissue DNA. The allele frequency was calculated as described in Materials and Methods.
